# A Review of Use of Enantiomers in Homeopathy

**DOI:** 10.5402/2012/575292

**Published:** 2012-08-15

**Authors:** R. M. Kuzeff

**Affiliations:** National Institute of Integrative Medicine, 759 Burwood Road, Hawthorn East, Melbourne, VIC 3123, Australia

## Abstract

This paper reviews publications of laboratory experiments using pairs of enantiomers in homeopathy. Many molecules in nature have geometry which enables them to exist as nonsuperimposable mirror images or enantiomers. Modulation of toxicity of such molecules provides possibility for therapeutics, since they target multiple points in biochemical pathways. It was hypothesized that toxicity of a chemical agent could be counteracted by a homeopathic preparation of the enantiomer of the chemical agent (patents applied for: PCT/AU2003/000219-PCT/AU2008/001611). A diverse body of data, including controlled laboratory studies, supports the conclusion that toxicity of optical isomers may be inhibited by homeopathic enantiomer preparations. These data were obtained with minimal or no pretesting to determine optimal test solutions. Inhibition of the excitotoxic neurotransmitter L-glutamic acid with homeopathic preparations of D-glutamic acid indicates the latter may be of use for amelioration of symptoms of disturbances of mood. Similarly, homeopathic preparation of (+)-nicotine may be of use for inhibition of effects of nicotine in tobacco.

## 1. Introduction

This paper discusses laboratory research involving the modulation of optical isomer (optical isomer: a molecule that is not superimposable on its mirror image) activity by use of homeopathic (potentized (Potency: A medicinal preparation used in homeopathy which has been prepared by serial dilution with succussion, trituration, or other form of agitation, e.g., sonication, between each stage of dilution.)) preparations of their enantiomers (enantiomer: either of a pair of optical isomers that are mirror images of each other). These experiments constituted the first time such stereochemical considerations had been tested in homeopathic research [1]. This was confirmed by examination at the Australian, US, and European patent offices in relation to PCT/AU2003/000219 [2]. Work using enantiomers in homeopathy has been published mainly in patents. Five experiments have also been published in 4 journal articles [3–6] with the balance available on line at WIPO [2, 7] and the United States Patent Office web site [8]. The experiments were performed in Bulgaria, Hungary, and Scotland.

The notion of treating the effects of optical isomers by use of their enantiomers in homeopathy first arose in the mid 1980s, not long after investigators published their first paper regarding isopathic use of pollen in treatment of hay fever [9]. Isopathy is the treatment of a disease caused by a morbific agent, by using a potency of the identical morbific agent [10]. At about that time the author of the instant paper noted that d-tubocurarine, a nondepolarizing neuromuscular blocker used for muscle paralysis during surgery was an optical isomer. He had toyed with the idea of using simple similarity in homeopathy to inhibit action of neuromuscular blockers, that is, using a homeopathic preparation of a neuromuscular blocker to modulate the toxicity of another neuromuscular blocker, for example, using alcuronium in pharmacological doses for neuromuscular blockade and administering a homeopathic potency of pancuronium or atracurium in an attempt to inhibit the toxicity of alcuronium. It seemed remarkable that ultrahigh dilutions attenuated beyond Avogadro's number may be biologically active, although not all homeopathic preparations are ultradilute. In theory ultradilute solutions do not contain even a single molecule of the original medicinal substance from which the preparation is derived.

In 1990 the author completed a homeopathic course, and after a period of trying to apply homeopathic principles in the clinic, he was ready to give up homeopathic practice. Although there did seem to be useful and even dramatic effects here and there that were hard to explain as placebo, the outcomes were too inconsistent in his hands to be useful in practice. The main problem in classical homeopathic prescribing was determining the appropriate medicine on the basis of the symptoms and signs of the patient. It was difficult to translate the presentation of the patient into the language of homeopathy such that a correct prescription could be given. It was clear also from clinical meetings that this was the problem other practitioners faced. Issues of which potency should be attempted were resolved very quickly in meetings and ultimately determined empirically. The single most complex and time-consuming step by far was the determination of an appropriate medicinal substance.

The author then read the first three books by Sankaran on theory and practice of homeopathy as understood by Sankaran [11–13]. He was thereby sufficiently inspired to give homeopathy one last go in his practice, in the form of a prospective double-blind randomized placebo-controlled study [14]. The author was further inspired by the fact that if a study is prospective, double-blind, randomized and placebo controlled, then it is scientifically and mathematically prudent to accept that the observed difference is valid. The results of the author's randomized controlled trial (RCT) performed in his clinic, and which measured a visual analogue scale (VAS) of sensation of well being and CD4 lymphocyte levels, were sufficiently encouraging to warrant seeking an easily reproducible laboratory method for detection of homeopathic effects, that would not require the expensive labour intensive input of a clinical study.

Many molecules in nature have geometry which enables them to exist as nonsuperimposable mirror images or enantiomers. Modulation of toxicity of such molecules provides the possibility for therapeutics, since these molecules target multiple points in biochemical pathways, although it must be admitted that use of potentized substances in this way may be anathema to the principles of homeopathy which involves a number of steps in case analysis and a particular philosophy. It was hypothesized that toxicity of a chemical agent may be counteracted or modulated by a homeopathic or potentized preparation of the mirror image of the chemical agent [2, 3, 7, 8] (Australian patent no. AU2003208170, Australian patent applications 2010203124 and 2009201723, Indian patent application no. 2704/DELNP/2004, Indian patent no. 246566, European patent application no. EP1487448, US patent no. 8,071,630, US patent application 13/304,618, Canadian patent application no. CA 2505984, Hong Kong patent application no. 05105229.7, New Zealand patent numbers 535654 and 588787). It was speculated that such use of enantiomers for modulating optical isomer toxicity may mimic the simillimum principle of homeopathy better than, say, the principle of isopathy [3].

The simillimum principle is one of the basic principles of homeopathy. According to this principle, the curative homeopathic remedy is the one that produces the set of symptoms most like that which the disease produces; ideally the disease symptoms and those of the remedy are exactly congruent. This should not be confused with isopathy where something “identical” to the morbific agent is used rather than something which is “similar”.

The hypothesis espoused in the previous paragraph may seem a bold one; however, homeopathic practitioners have asserted the importance of the simillimum principle for over 200 years. According to most practitioners the selection of the correct medicine (simillimum) is of greater importance than potency selection [15] and, by implication, method of manufacture. The only selection criterion for use of any of the compounds in this paper was that the molecules in question possessed at least one chiral centre (or stereogenic carbon atom).

## 2. What Is an Optical Isomer?

**Figure 1:**
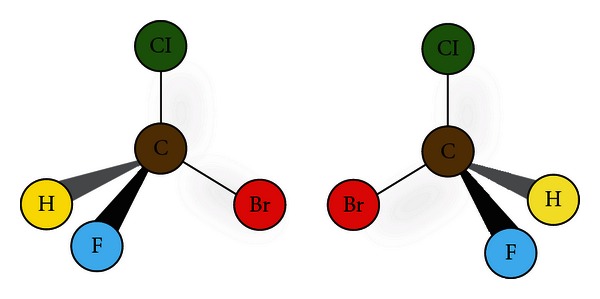
Reference [17] this shows a simple example of an optical isomer. Optical isomers are not superimposable on their mirror images. The 2 molecules depicted above are mirror images of each other, and they cannot be superimposed in 3-dimensional space.

Figure 1 depicts a simple example of an optical isomer. The most common cause, although not the only cause of optical isomerism, is the presence of one or more stereogenic carbon atoms (chiral centres) in a molecule [16]. Such a carbon atom is characterized by the fact that it is bound to 4 different groups or substituents. In Figure 1 the substituents are hydrogen, chlorine, bromine, and fluorine; however, one could replace these substituents with any other structure including, but not limited to, amine groups, carboxyl groups, or any other hydrocarbon side chain. Less commonly phosphorus, nitrogen and sulphur may form chiral centres, as may transition metals in coordination compounds.

## 3. What Is Homeopathy?

Homeopathy is a system of medicine that rests upon 5 main principles [18]:

- the Law of Similars (simillimum principle)
- the principle of potentization
- the principle of the minimum dose
- the principle of the totality of symptoms
- Hering's Law of the direction of cure.

The best known of these to nonpractitioners would be the Law of Similars, also referred to as *similia similibus curentur* or *let likes be cured by likes.* Note that it does not say “identical cures identical.” This latter expression would conform with the principle of isopathy.

In essence this means to cure a patient, a medicinal substance or agent must be used which is capable of producing the symptoms from which the patient is suffering. However, a simple similarity is not intended by item (i), mentioned previously. For an agent to be correctly selected it should ideally be capable of producing all the symptoms of the patient and not just one. This is the principle of the totality of symptoms. Thus, item (i), mentioned previously, should always be used in conjunction with item (iv).

## 4. How Are Homeopathic Medicines Prepared?

**Figure 2:**
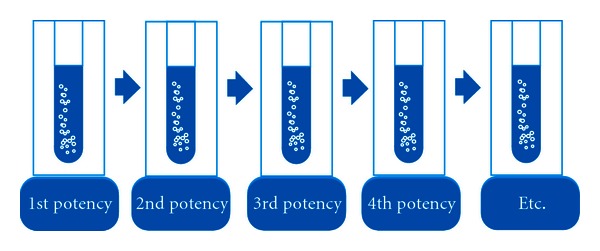
In the decimal system of attenuation each test tube shown above would contain 1/10 the quantity of medicinal substance as the preceding test tube. Each stage of dilution is referred to as a potency. The same principle applies for centesimal potencies.

Homeopathic medicines are typically produced by a process of serial dilution and succussion, and historically this has been done manually; however, automated devices have been used as has sonication or other form of mechanical agitation such as use of a Lab Dancer. Trituration may also be used, but this discussion will restrict itself to preparation of liquid homeopathic potencies as depicted in Figure 2. Preparation of medicines is usually done in so-called decimal and centesimal scales; that is, each successive attenuation contains 1/10 or 1/100 as much of the medicinal substance as the preceding attenuation, typically starting with 1/10 or 1/100 by weight or volume of medicinal substances in the preparation of the first potency. At each stage of attenuation the test tubes would be succussed typically between 10 and 100 times [2]. Such successive attenuations are referred to as potencies. The fifty millesimal system is another standard scale of attenuation.

As serial dilution and succussion proceeds, there comes a point where the level of dilution of the original medicinal substance exceeds Avogadro's number (6.022 × 10^23^). After this point it becomes progressively more unlikely that the succeeding potencies will contain even a single atom or molecule of the original medicinal substance. Such potencies are ultradilute. In the case of decimal potencies this occurs from the 24th potency onwards. In the case of centesimal potencies, this occurs from the 12th potency onwards. It should be remembered that a potency only becomes homeopathic when it is administered according to the principles of homeopathy listed previously. The various methods which are sometimes collectively called homeopathy and are described by Watson [10] are distinct and diverse practices and should not be included simultaneously in meta-analyses of the literature because lack of homogeneity of studies precludes rigorous meta-analysis.

## 5. Experiments Using Enantiomers in ****Homeopathy

In order to test the null hypothesis that potentized optical isomers were no different from placebo in terms of their ability to modulate toxicity of their enantiomers, experiments were done in 3 countries: Bulgaria, (Bulgaria: Topashka-Ancheva MN, Metcheva RP. Institute of Zoology, Bulgarian Academy of Sciences), Hungary (Hungary: Gruiz K. Molnar M. Hadjú C. Tolner M. Budapest University of Technology and Economics), and Scotland (Scotland: Kozlova-Zwinderman O. Christofi N. Lutess Ltd, Edinburgh). Experiments used a number of designs including blind, randomized, controlled experimental studies and controlled laboratory studies [2–8, 19–21]. A feature of the experiments discussed in this paper was that they were performed with minimal or no pre-experimentation to determine optimal potencies, methods of manufacture, or starting concentrations of medicinal substance and that diverse species and enantiomers worked within the hypothesis; further, in spite of the diversity of compounds, species and methodologies used, all experiments were prospective tests of the same null hypothesis. Experiments 1–26 inclusive shown later are actually 26 experimental series, each consisting of between 1 and 4 experiments.

### 5.1. Experiments with ICR Mice

Experiments were done using ICR mice at the Institute of Zoology, in Sofia Bulgaria [3–6, 21]. Except for the first experiment, which was an unblinded randomized placebo-controlled pilot study, mice in the remaining experiments were randomized blind into control and treatment arms. These arms were treated, respectively, with indistinguishable control and potency solutions. The optical isomers were injected intraperitoneally, at a dose sufficient to kill 50% of the control group mice (LD50). The number of mice surviving the procedure was the unequivocal endpoint of the experiment. The number surviving in the control and treatment groups was subjected to statistical analysis. Throughout the work on optical isomers in homeopathy, varying methods of potency preparation were used in order to see if the hypothesis was robust. Experiments 1–5 listed in the following were done at the Institute of Zoology at the Bulgarian Academy of Sciences between 2002 and 2009.

**(1) Prospective Randomized Placebo-Controlled Pilot Study Testing Inhibition of Toxicity of (−)-Propranolol Hydrochloride by a Homeopathic Potency of Its Enantiomer in ICR Mice [3]**

The first experiment was a pilot study using Propranolol HCl in ICR mice to test the experimental methodology and to see if the toxicity of (−)-propranolol HCl may be inhibited by a homeopathic (i.e., potentized) preparation of (+)-Propranolol HCl. A 30th potency was used which represented a notional concentration of 3.4 × 10^−62^ M (notional concentration: this terminology is used when ultrahigh dilutions are mentioned in this paper, since such ultrahigh dilutions theoretically contain none of the original solutes, and as such concentration is zero). Note that 30th potency was prepared fresh from 29th potency on each day of the experiment. Design was prospective, unblinded, randomized and placebo controlled. Mice were randomized into equal-sized treatment and placebo arms each containing 77 mice for a total of *n* = 154. 12% more treatment mice survived than placebo mice. Since LD50 was being used, the maximum superiority of survival in the treatment group would have been 50%. Results were *P* = 0.1481, OR 1.64 by 2-sided chi-square test. 30/72 (42%) of the mice treated with the (+) stereoisomer potency were alive approximately 23 hours after receiving the ip injection, compared with 23/76 (30%) of those receiving indistinguishable placebo. Exclusions were determined by prospective criteria due to death of mice after administration of anaesthetic agent, but before administration of treatment and placebo fluids.

Since we knew that smaller mice had a higher metabolic rate and anticipated that this would affect propranolol action and elimination [22], the data were adjusted for weight by logistic regression. This yielded *n* = 154, *P* = 0.089, OR 2.02, 95% confidence interval (CI) of 0.90–4.53 for a 2-sided test. Although not significant at the traditional 5% level, these data were indicative of a trend to significance. Accordingly a power calculation was performed to determine what sample size may be needed to achieve statistical significance at the 5% level. Assuming that the real difference in survival was between 9% and 24%, a sample of approximately 500 mice was required : 250 treatment and 250 placebo, assuming *P* = 0.05 with 17% improved survival in the treatment group, that is, 50% survival in the treatment group and 33% survival in the placebo group, gave a power of 0.95 for a two-sided test and 0.98 for a one-sided test [3].

**(2) Prospective Blind Randomized Placebo-Controlled Laboratory Study Testing Inhibition of Toxicity of (−)-Propranolol Hydrochloride by a Homeopathic Potency of Its Enantiomer in ICR Mice [4]**

This was the study performed after experiment 1, previously. This study yielded *P* = 0.0247, OR 1.52 by chi-square, *n* = 508 for a 2-sided test. By logistic regression adjusting for mouse weight, *P* = 0.0283, OR 1.51, 95% confidence interval (CI) of 1.045 to 2.19, by 2-sided test. The study was identical to that described in 1, previously, except that a larger sample was used, and only male mice were admitted in order to increase homogeneity of the sample. Another difference was in the preparation of the 30th potency which was used as treatment in the experiment. In this experiment 117/238 (49%) of the mice treated with the (+) stereoisomer potency were alive approximately 23 hours after receiving the ip injection, compared with 96/246 (39%) of those receiving indistinguishable placebo. Exclusions were again determined by prospective criteria. Sixteen treatment mice died shortly after administration of Rometar anaesthetic compared with 8 placebo mice. Interestingly, this showed a trend to significance according to chi-square test (*P* = 0.0943) and Fisher exact test (*P* = 0.1418) for a 2-sided test. This outcome therefore suggests that pretreatment with the potentized isomer may increase the subsequent toxicity of Rometar in mice. The 30th potency in this experiment represented a notional concentration of 2.4 × 10^−59^ M.

**(3) Prospective Blind Randomized Placebo-Controlled Laboratory Study Testing Inhibition of Toxicity of (−)-Trans-(1S,2S)-U-50488 HCl by Its Enantiomer in ICR Mice [5]**

This experiment was identical to experiment 2, except that it investigated the ability of a (+)-U50488 HCl potency chord to inhibit the toxicity of (−)-U50488 HCl injected intraperitoneally. U-50488 is a potent kappa opioid agonist [23]. As discussed in a previous paper [4], the instant experiment was considered a purer test of the hypothesis than the preceding experiments using propranolol, since the toxic symptoms of the mice were not confused by simultaneous administration of Xylazine (Rometar) as an anaesthetic agent. Symptomatology is critical in homeopathy. Accordingly, in the present experiment, one may anticipate larger between group differences in the primary endpoint if the null hypothesis is incorrect.

A potency chord is a mixture of different potencies of the same substance in dosage form. In this study, the potency chord consisted of a mixture of 4th, 12th, and 30th potencies of U-50488 in equal quantities and represented a U50488 concentration of 1 × 10^−10^ M. The method of potency preparation is described in the paper and was arbitrary once again in order to test robustness of the hypothesis.

53/79 (67%) of the mice treated with the (+)stereoisomer potency were alive approximately 23 hours after receiving the ip injection, compared with 37/78 (47%) of those receiving indistinguishable placebo. There was a single exclusion according to prospective criteria in a placebo mouse due to sudden death after intraperitoneal injection of Rometar injection. Results showed improved survival in the treatment group with *n* = 158, *P* = 0.0128, by chi-square. Logistic regression with adjustment for mouse weight as performed in the previous mouse studies yielded *n* = 158, OR 2.30, *P* = 0.0128, 95% confidence interval (CI) 1.194–4.434.

**(4) Two Prospective Blind Experimental Studies Comparing the Isopathic versus Enantiomeric Inhibition of (−)-Trans-(1S,2S)-U-50488 HCl in ICR Mice [6]**

This paper contained two blind randomized studies. Their designs used the template of experiment 3. The first study investigated the ability of a chord of 4th, 12th, and 30th potencies of (−)-U-50488 HCl in terms of its ability to inhibit the toxicity of (−)-U-50488 HCl injected at the LD50 dose intraperitoneally into 158 male ICR mice. This was an isopathic study. Isopathy is well known in homeopathy [10]. There was no significant effect of isopathy (chi-square = 0.9215, 1 degree of freedom, *P* = 0.3771, *n* = 158). There was a 3% nonsignificant improvement in survival in the isopathic treated group compared with the placebo group.

The second study was performed on 300 male ICR mice. Design was a prospective, blind, randomized equivalence study. It compared the action of potencies of the (+)- and (−)-enantiomers of U-50488, in terms of their ability to inhibit toxicity of (−)-U-50488. According to the null hypothesis, there should be no between group difference, since both the preparations were homeopathic potencies and accordingly should have no effect. It was shown that the (+)-isomer of U-50488 HCl in the form of a potency chord was significantly more effective than an identically produced isopathic potency chord of (−)-U-50488 HCl in terms of inhibiting the toxicity in mice poisoned with (−)-U-50488 HCl. In both these studies, the potency chords used were a mixture of 4th, 12th, and 30th potencies of the respective enantiomers.

Potencies were prepared as per previous U-50488 experiments and represented concentrations of 1.08 × 10^−10^ M. The mice treated with a potency chord made from (+)-isomer were 1.97 times more likely to survive than mice treated with a potency chord made from the (−)-isomer of U-50488 which is the isopathic preparation. The *P*-value in favour of survival of the (+)-isomer potency compared with the (−)-isomer potency (isopathic treatment) was *P* = 0.0047 (95% CI: 1.23–3.14). 17% more mice survived in the group that was treated with enantiomeric potency made from (+)-U-50488 than in the isopathically treated group with potency made from (−)-U-50488.

Isopathic experiments using optical isomers have been previously reported [24–28]. Jonas et al. reported effects of potencies of (−)-glutamate isopathically inhibiting (−)-glutamate toxicity in the nervous system of rats [26]. Bonamin et al. reported significant inhibition of dexamethasone activity by administration of isopathic potencies of dexamethasone in BALB/c mice [24]. To treat the toxicity of intraperitoneal injections of (−)-U-50488 in an isopathic way, one would use potencies of (−)-U-50488. When the enantiomer is used to treat the toxicity of (−)-U-50488, that is, (+)-U-50488, then this represents an example of enantiomeric, chiral, or stereoisomeric treatment in homeopathy.

Stereospecificity and stereoselectivity dictate that the pharmacokinetics, pharmacodynamics, physiological actions, and therefore symptoms produced by stereoisomers may differ from one another [29, 30]. Accordingly, it is misleading to think of enantiomeric treatment as being semi-isopathic, since in isopathy, the effects of a morbific agent are treated with a potency of the same agent. Also, as demonstrated in this study, experiment 4, and the replication thereof in experiment 5, the effects of enantiomeric treatment are different from isopathic treatment.

**(5) Prospective Blind Experimental Study Comparing the Isopathic versus Enantiomeric Inhibition of (−)-Trans-(1S,2S)-U-50488 HCl in ICR Mice [21]**

This experiment was a replication of the 300 mouse experiment described in experiment 4. Once again survival in the enantiomerically treated group was superior to the isopathic group. Treatment with (+)-U-50488 potency compared with (−)-U-50488 potency gave a *P*-value of 0.0117 by chi-square test with OR (+ versus −) of 1.7902 (95% CI: 1.1367–2.8193) for a two-sided test and a *P*-value of 0.0124 by logistic regression which adjusted for mouse weight with OR 1.34 (95% CI: 1.07–1.68). These results are consistent with the statistical ranges determined in experiment 4. Comparing results of the logistic regression analyses from the enantiomer studies in experiments 4 and 5, in the first study, 99/150 (66%) of the mice treated with the (+) potency were alive 23 hours after receiving the ip injection, compared with 74/150 (34%) of the mice receiving the (−) potency. By comparison, in the present study 93/153 (61%) of the mice treated with the (+) stereoisomer were alive approximately 23 hours after receiving the ip injection, compared with 71/153 (46%) of those receiving the (−) stereoisomer. Potencies had U50488 concentrations of 1.08 × 10^−10^ M.

### 5.2. Experiments with *Vibrio fischeri*, *E. coli*, *Pseudomonas fluorescens*, *Tetrahymena pyriformis*, and *Daphnia magna*

Experiments 6–18 were undertaken in Budapest using various species, in another attempt to prove the null hypothesis. In these experiments organisms were poisoned with the neurotransmitter L-glutamic acid (L-GA) to investigate if homeopathic potencies of its enantiomer, D-glutamic acid (D-GA), were able to modulate toxicity in accordance with the hypothesis. Similarly, experiments were done using nicotine. In the glutamic acid experiments which follow potencies of D-glutamic acid were made by adding 10 mg D-glutamic acid to 1 mL water followed by succussion to produce the first potency. Subsequent potencies were made by serial dilution by a factor of 1 : 250 followed by succussion [7]. This was another arbitrary scale of attenuation.

**(6) Glutamic Acid Experiment Investigating the Treatment of Toxic Effects of L-Glutamic Acid with D-Glutamic Acid Potencies Using the Vibrio fischeri Bioluminescence Test [31]**

The *Vibrio fischeri* bioluminescence test is a well-known acute toxicity test [32]. The test is further described in experiment 17. It uses the luminescent bacterium *Vibrio fischeri* and measures the light emission of the bacteria after toxic challenge, which in this case was by L-glutamic acid, and compares the result with a blank control. The difference in light emission between sample and control is due to the effect of D-glutamic acid potency. EC_20_ and EC_50_ values were determined from concentration-response curves after sigmoidal fitting by ORIGIN 6.0 software. 4th, 15th, and 30th potencies were made according to protocol [7, 31]. Results showed significant decrease at the 5% level in toxicity in the organisms treated with 4th and 15th potencies of the enantiomer D-glutamic acid compared with placebo and no effect with the 30th potency of D-glutamic acid.

**(7) Treatment of Toxicity of L-Glutamic Acid by Its Potentized Enantiomer in E. coli: No Pretreatment Experiment [7, 33]**

In *E. coli* and then *Pseudomonas fluorescens*, 5th, 13th, and 31st potencies were used due to a misunderstanding. It had been intended to test 4th, 12th, and 30th potencies and also a potency chord consisting of equal parts of 4th, 12th, and 30th potencies. In spite of this, numerous results were obtained supportive of the method of using enantiomers in homeopathy. This is consistent with the homeopathic stance that the single most important factor in treatment is selection of an appropriate medicinal agent. In the case of *E. coli*, and unlike *Pseudomonas*, absorbance (represented by ODU) was not a suitable endpoint in cultures. The optical density values and the cell numbers were not proportional to each other. Instead, living cell concentrations were measured at 10 mg/tube L-GLU concentration under the microscope. In these experiments *E. coli* cultures were treated with placebo or potency, and cell numbers were then determined.

Results showed a difference in favour of treatment in the numbers of *E. coli* surviving in the treatment groups compared with the placebo groups for 5th potency, and this difference is between 13 and 500 times in absolute number. Thirteenth, 31st, and potency chords did not demonstrate effect. Due to lack of proportionality between optical density and E.Coli cell numbers, this was a suboptimal model for testing the action of these glutamic acid potencies. In this experiment the 5th potency had a D-GA concentration of 1.74 × 10^−11^ M, the 13th potency had a notional concentration of 1.14 × 10^−30^ M, and the 31st potency had a notional concentration of 7.84 × 10^−74^ M. The potency chord had a concentration of 1.74 × 10^−11^ M. Due to the way in which this potency chord was made, the dilution of D-GA was the same as for the 5th potency.

**(8) Treatment of Toxicity of L-Glutamic Acid by Its Potentized Enantiomer in E. coli [7, 33]**

In addition to the experiments noted in 7, and with the same reservations, experiments were conducted whereby cultures of both arms were pretreated with potency chord of 4th, 12th, and 30th potencies, and then subsequently treated with placebo or 5th, 13th, 31st potencies or potency chord, and results were compared. Once again 5th potency was reported as having a significant effect. Also, pretreatment with potency chord prior to administration of L-glutamic acid toxin, followed by further administration of potency chord, also showed superior survival in the treatment group compared with the placebo group. Once again, 13th and 31st potencies did not yield an effect. The concentrations of D-GA potencies used are the same as in experiment 7.

**(9) Treatment of the Toxic Effects of L-Glutamic Acid with D-Glutamic Acid Potencies in Pseudomonas fluorescens: No Pretreatment [7, 33]**

The concept of the tests on *Pseudomonas* was the same as in *E. coli. Pseudomonas* was cultured in multiple 100 mL flasks containing 30 mL nutrient medium under controlled conditions. After addition of *Pseudomonas* inocula to the nutrient media flasks, a series of varying concentrations of L-glutamic acid was made by adding 0, 2.5, 5, 10, or 25 mg L-glutamic acid to each of two parallel flasks. L-GA is toxic to *Pseudomonas*. After addition of L-GA to the flasks, a 500 *μ*L bolus of D-GA potency was added to one of the flasks, and a 500 *μ*L bolus of indistinguishable placebo was added to the parallel flask containing the identical concentration of L-GA.

**Figure 3:**
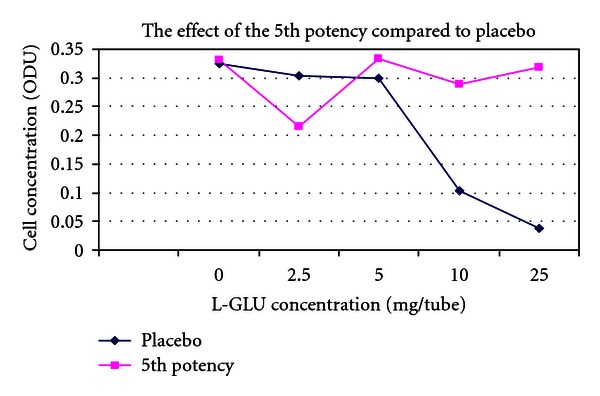
The effect of 5th potency D-glutamic acid potency versus placebo.

**Figure 4:**
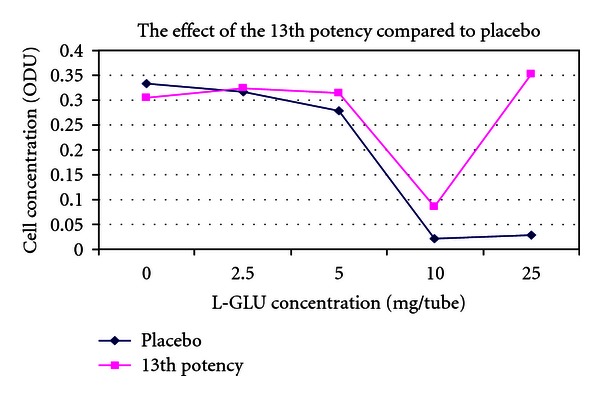
The effect of 13th potency D-glutamic acid versus placebo.

**Figure 5:**
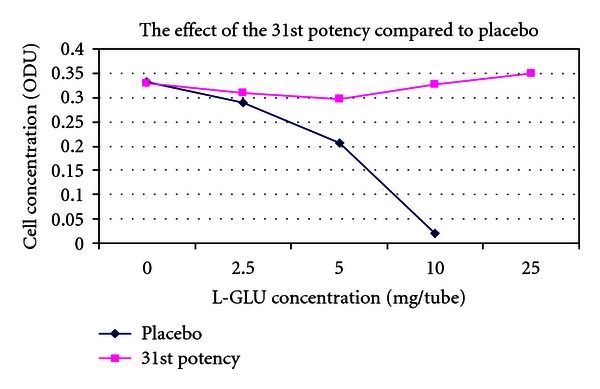
The effect of 31st D-glutamic acid potency versus placebo.

**Figure 6:**
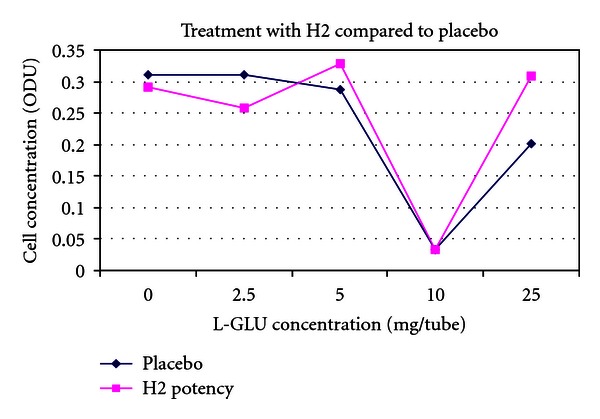
The effect of H2 (potency chord) D-glutamic acid versus placebo.

5th and 31st potencies (Figures 3 and 5) produced a dose-dependent response consistent with a protective effect of potency in L-GA toxicity compared with controls and would encourage further testing, whereas 13th potency and potency chord data did not. Thirteenth potency data and potency chord data (Figures 4 and 6) were not impressive.  Figure 4 shows one of the two potency chords which produced no evidence of effect (the other being in *E. coli* in experiment 8). Of interest is that all the cultures in Figures 3–6 showed increased survival in the treated group compared with placebo at the highest concentration of L-GA (25 mg/mL). 5th, 13th, and 31st potency D-GA concentrations are the same as in 8. The potency chord concentration of D-GA was 5.3 × 10^−12^ M.

**(10) Treatment of the Toxic Effects of L-Glutamic Acid with D-Glutamic Acid Potencies in Pseudomonas fluorescens: Pretreatment Followed by Posttreatment [7, 33]**

**Figure 7:**
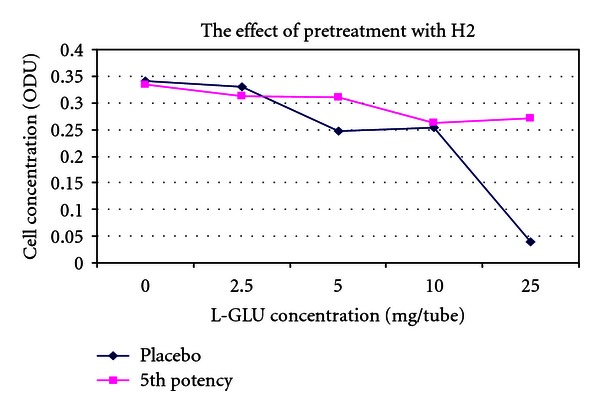
Effect of L-glutamic acid on H2 (potency chord) pretreated *Pseudomonas fluorescens* after 5th potency and placebo application.

**Figure 8:**
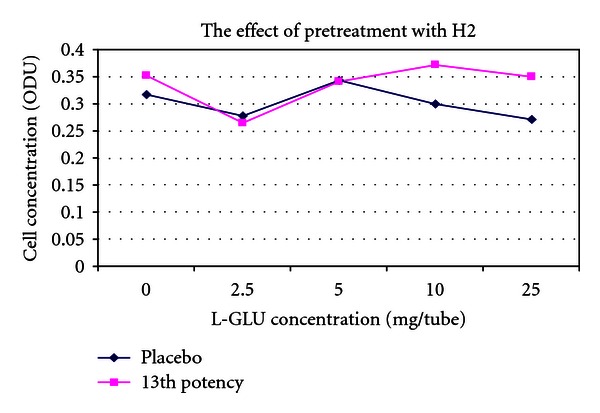
Effect of L-glutamic acid on H2 (potency chord) pretreated *Pseudomonas fluorescens* after 13th potency and placebo application.

**Figure 9:**
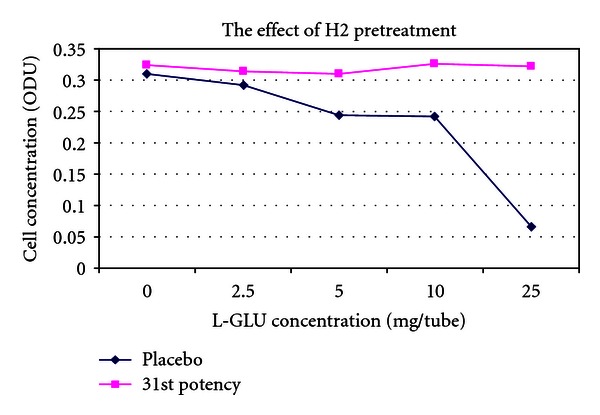
Effect of L-glutamic acid on H2 (potency chord) pretreated *Pseudomonas fluorescens* after H2 potency chord and placebo application.

**Figure 10:**
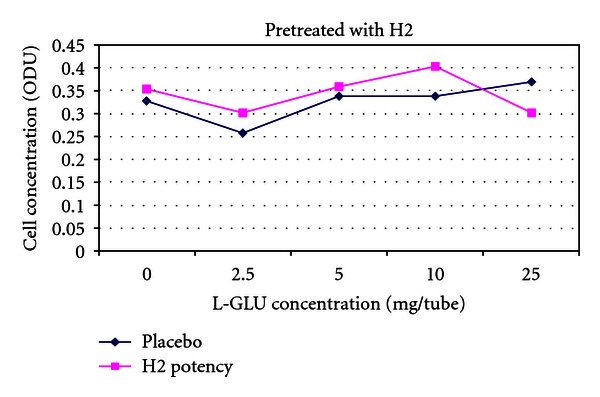
Effect of L-glutamic acid on potency chord (H2) pretreated *Pseudomonas fluorescens* after H2 potency chord and placebo application.

These assays were the same as in 9, above, except that prior to administration of *Pseudomonas* inoculum to the culture medium, each flask received 2.5 ml D-GA potency chord. In other words there was pre-treatment of both arms with potency chord consisting of equal parts of 4th, 12th and 30th potencies, and after administration of L-GA toxin, the 2 arms were respectively treated with placebo or more potency chord. Subsequently the placebo arms received a dose of placebo after administration of L-GA toxin, and the treatment arms received a dose of D-GA potency after administration of L-GA toxin. 5th, 13th, 31st and potency chord concentrations of D-GA are the same as for experiments 7 and 9. Results are shown in Figures 7–10.

Figure 7 is a pretreated version of Figure 3. The placebo line has moved higher up the *y*-axis and has converged with the treatment line. Considering that in this graph the placebo arm received pretreatment with potency, this outcome is not surprising and is consistent with an action of pretreatment on the placebo.

Figure 8 is a pretreated version of Figure 4. One can see that both lines on the graph have been shifted higher up the *y*-axis and have converged consistent with action of pretreatment.

Figure 9 is a pretreated version of Figure 5. One can see that both lines on the graph have converged consistent with an effect of pretreatment.

Figure 10 is a pretreated version of Figure 6. One can see that both lines on the graph have moved up the *y*-axis and have converged which is consistent with an effect of pretreatment. Note that although Figure 6 produced no effect, when the same experiment used pretreatment with the same potency chord in Figure 10, the result was consistent with biological activity of the potency chord.

**(11) Treatment of the Toxic Effects of L-Glutamic Acid with D-Glutamic Acid Potencies in Tetrahymena pyriformis [7, 33]**

In these tests the effect of 13th potency D-glutamic acid (notional concentration D-GA 1.14 × 10^−30^ M) was tested in terms of its ability to modulate the toxicity of L-glutamic acid, in the protozoan *Tetrahymena pyriformis*. The preparation of potency was the same as for the experiments with *Pseudomonas*. The potency used was diluted beyond Avogadro's number, and therefore it is unlikely that the dilution contains even a single molecule of D-glutamic acid unless recent work reported by Chikramane et al. is correct; however, it is uncertain how these findings in metals can be extrapolated to organic starting materials [34]. Further, the Chikramane findings are relevant if one samples from the top of the fluid in a test tube; this would be an unusual and awkward practice for the production of homeopathic medicines in our experiments, making it difficult to avoid sucking up air into the pipettes during preparation of test solutions.

**Figure 11:**
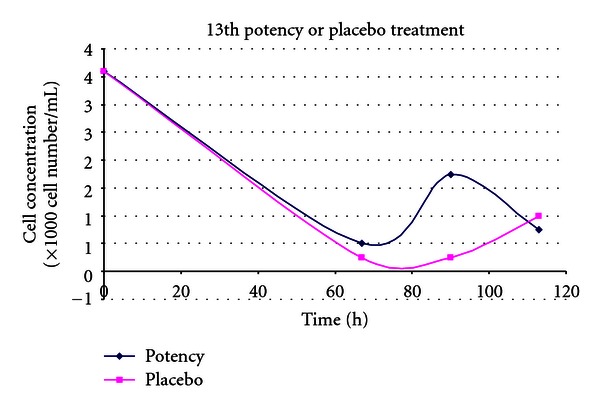
Effect of 13th potency D-GA compared with placebo on *Tetrahymena pyriformis* poisoned with 15 mg/30 mL L-GA. *Tetrahymena* were grown in culture medium for 24 hours before addition of L-glutamic acid as toxin.

In the first experiment shown in Figure 11, *Tetrahymena* cultures were grown for 24 hours prior to administration of 0.5 mg/mL L-glutamic acid to each culture. Ten minutes after addition of the L-GA toxin, a bolus of placebo or 13th potency D-GA was added to the placebo and treatment cultures, respectively. Figure 11 shows that the graph line depicting the culture treated with 13th potency of D-GA added to culture was consistent with less inhibition in *Tetrahymena* growth after 60 hours of incubation. In the tests with *Tetrahymena* very few doses of potency were used considering the duration of the assays (5 days).

The timing of potency administration in the next experiment was changed in order to elicit more convincing evidence of potency effect than was the case in experiment 11.

**(12) Treatment of the Toxic effects of L-Glutamic Acid with 13th Potency D-Glutamic Acid Potencies in Tetrahymena: Pretreatment Experiment [7, 33]**

The second trial involving *Tetrahymena* like the first involved propagation in 30 mL flasks. Rather than adding 13th potency D-glutamic acid to the cultures after 24-hour incubation, *Tetrahymena* was exposed to D-GA potency or placebo 24 hours prior to addition of the *Tetrahymena* inoculum to the flasks. Subsequently 15 mg/flask or 0.5 mg/mL L-GA was added to the treatment and placebo arms, and 10 minutes thereafter another bolus of treatment or placebo was added to the respective arms. This contrasts with experiment 11, where *Tetrahymena* inocula were added to culture medium and grown for 24 hours before L-glutamic acid was added to the flasks.

**Figure 12:**
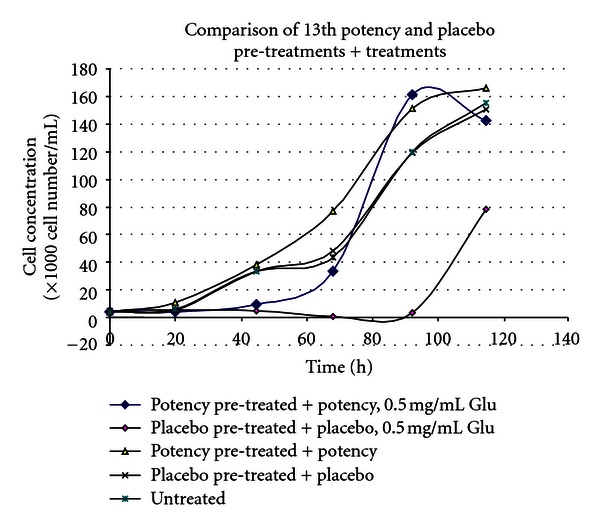
Propagation in 30 mL flasks, prevention type pretreatment with 13th potency in 1/10 volume ratio, curative treatment with 13th potency in 1/60 volume ratio: all combinations, that is, 1 part potency or placebo in 60 parts culture. See description below for clarification. Unlike Figure 11, *Tetrahymena *inocula were exposed for 24 hours to 13th potency D-GA potency or placebo, before being placed in flasks with culture medium. L-glutamic acid was added to the culture flasks 10 minutes later.

The lines in Figure 12 represent cultures which had varying interventions. The first line in the key (*potency pretreated *+* potency, 0.5 mg/mL Glu*) had treatment (prevention type pretreatment) with D-GA potency 24 hours before addition of L-GA toxin. The culture then had further potency added 10 minutes after addition of L-GA (curative treatment). The second line represents the scenario with addition of indistinguishable placebo pretreatment followed by L-GA toxin and then further placebo 10 minutes thereafter. The remaining lines represent 3 cultures which did not have L-GA added to them and are types of controls. In view of what has just been said they should be self-explanatory. The arm which received 13th potency D-GA 10 minutes after addition of L-GA, and also at 24 hours before addition of L-GA, showed less inhibition of growth due to L-GA compared with the identical placebo, which is the lowest graph line on the *y*-axis. The 4th and 5th lines on this graph show 2 types of placebos with very similar growth curves. They show the growth curve unaffected by L-GA toxicity.

The 3rd line shows the growth curve in the absence of L-GA toxicity, but with pretreatment with 13th potency administered 24 hours prior to addition of L-Glu (L-GA) toxin and also with addition of potency in parallel with the flasks which received L-GA or in other words 0.5 mg/mL L-GA. This is another type of control. This line is shifted up the *y*-axis compared with the other two controls and suggests that addition of this ultradilute potency may have actually stimulated *Tetrahymena* growth. In Figure 12, the L-GA poisoned control lies at the bottom of the graph consistent with action of L-GA toxin. The graph line immediately above this represents the culture which obtained treatment. One can see that improvement in survival in the treatment arm starts just before the 40-hour mark; by 95 hours, survival is well above the controls on the *y*-axis.

**(13) Treatment of the Toxic Effects of L-Glutamic Acid with 13th Potency D-Glutamic Acid Potencies in Tetrahymena: No Pretreatment [7, 33]**

This was a replication of experiment 12, but without 13th potency pretreatment. The explanation of the 5 different graph lines in the key is the same as in experiment 12.

**Figure 13:**
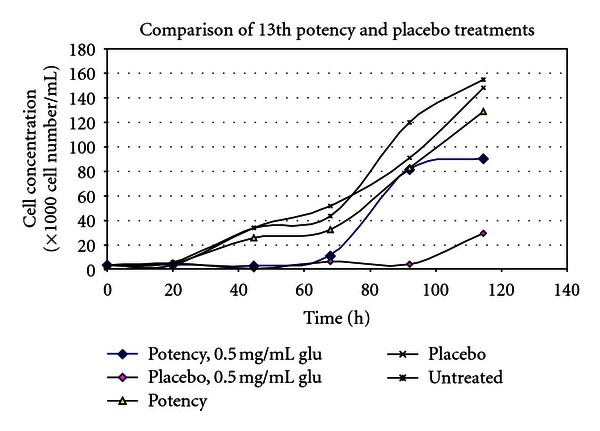
Identical experiment to that in Figure 12, except that there was no pretreatment with 13th potency.

Figure 13 shows a similar trend to that in Figure 12. The difference is that the inocula did not receive pretreatment with potency or placebo 24 hours prior to addition of the inocula to the culture medium in the flasks. Potencies and placebos were only added after addition of L-GA to the cultures. This time, both the placebo and treatment arms languish at the bottom along the *y*-axis of the graph, until they diverge at 63 hours demonstrating increasing survival of *Tetrahymena* in the treatment arm compared with the placebo arm. Note that experiment 12 which had additional pretreatment compared with experiment 13 showed higher survival of *Tetrahymena* organisms.

**(14) Treatment of the Toxic Effects of L-Glutamic Acid with 13th Potency D-Glutamic Acid Potencies in Tetrahymena: No Pretreatment [7, 33]**

**Figure 14:**
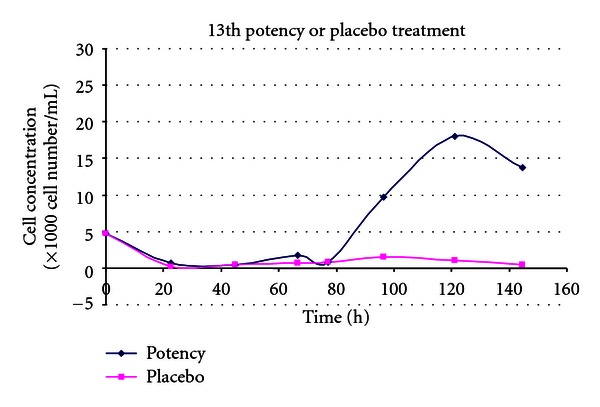
Simplified replication of the experiment depicted in Figures 12 and 13. Both arms were poisoned with 15 mg/30 mL L-glutamic acid. The graph shows culture treated with 13th potency D-glutamic acid versus Placebo.

Figure 14 shows a replication of the experiment in Figure 13 comparing placebo and treatment arms. *Tetrahymena* concentrations took more time than usual to start increasing and languished until the 75-hour mark. At that time the graphs diverge showing increased cell numbers in the treatment arm compared with the placebo arm. The outcome is consistent with the hypothesis.

**(15) Treatment of the Toxic Effects of L-Glutamic Acid with 13th Potency D-Glutamic Acid Potencies in Tetrahymena: No Pretreatment [7, 33]**

**Figure 15:**
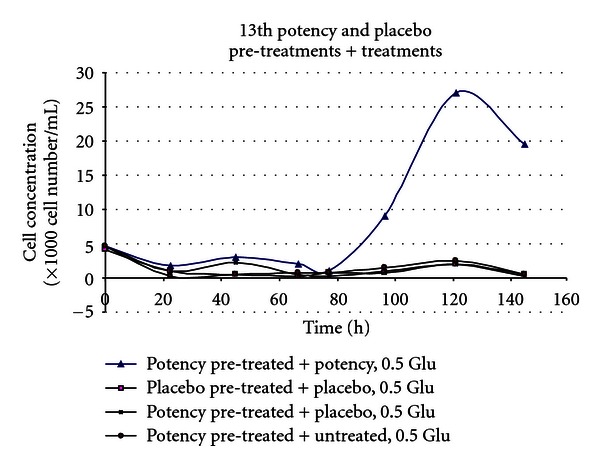
In this experiment potency pretreatment 24 hours before addition of L-GA had no effect on subsequent growth, although potency pretreatment followed by another bolus of potency after addition of L-GA did show improved survival.

Figure 15 shows the effect of D-GA potency on growth of *Tetrahymena* in cultures poisoned with L-GA 0.5 mg/mL. In this experiment all arms received 15 mg L-GA added to the culture flasks. The first line in the key shows the growth of *Tetrahymena* that had been pretreated with 13th potency D-GA, subsequently poisoned with 15 mg/mL L-GA and thereafter treated with a bolus of 13th potency D-GA. This was the only growth curve which showed increased survival of *Tetrahymena*. The second line in the key shows the culture which received placebo before and after addition of L-GA. The third line shows the growth line of the culture which was treated with potency before addition of L-GA and placebo after L-GA addition. The fourth line shows the growth of the culture which received potency before addition of L-GA and no treatment after addition of L-GA. This experiment is weak evidence of the effect, since it was not stratified. There are 3 treatment arms and 1 placebo arm. The arm which did demonstrate increased survival was the scenario of maximum treatment, that is, pretreatment with potency before administration of L-GA toxin, with additional treatment after administration of L-GA. As such, this experiment provides confirmation of the hypothesis. A replication with block design would be appropriate.

**(16) Morphology of Tetrahymena pyriformis Poisoned with L-Glutamic Acid [7, 33]**

**Figure 16:**
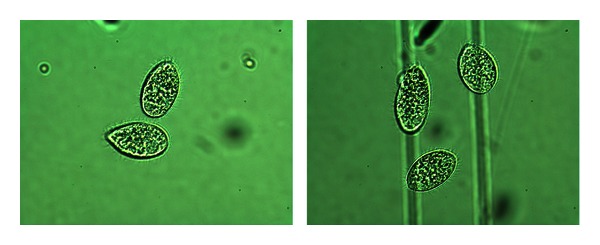
Normal *Tetrahymena pyriformis* cells. No L-glutamic acid was added to cultures.

**Figure 17:**
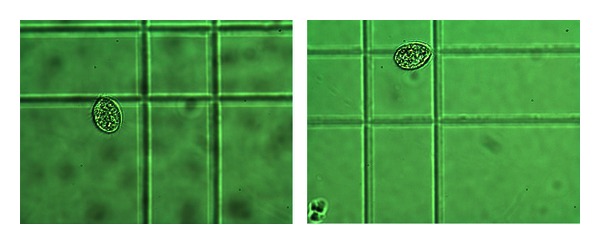
*Tetrahymena* poisoned with L-glutamic acid.

**Figure 18:**
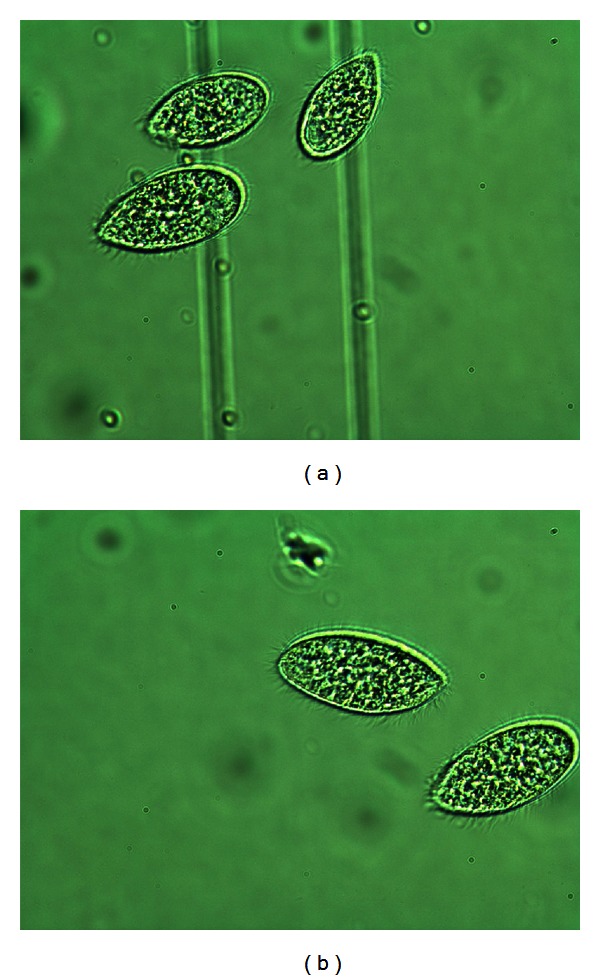
*Tetrahymena pyriformis* treated with potency (b) and pretreated in addition to receiving treatment with potency after L-GA addition (a).

In addition to the cell numbers some photographs of *Tetrahymena* were taken, because morphological differences between potency treated and placebo cultures we observed. See Figures 16, 17, and 18.

Normal cells in optimal culture medium in the previous 2 pictures, without any treatment: the cells appear big and healthy, moving intensively with their hair-like filaments. The size can be seen from the lines under the cells, which are the engravings of a Buerker-chamber, and shown on most of the pictures.

L-GA + placebo: the L-GA poisoned cells are not only fewer in number, but the surviving ones were much smaller than the healthy ones. Their inner structure is also different, with less structured morphology.

The picture on the right received D-GA potency after addition of L-GA 0.5 mg/mL to the culture. The picture on the left was given pretreatment with potency as well as treatment after L-GA addition to the culture. The latter had higher cell concentration than the former. Morphology was preserved in each case. You can compare the pictures according to the size: the same magnification was used for each. The sizes of the pictures are comparable by the engraving of the chamber. If the two lines are an identical distance apart, the magnification of the picture is the same.

**(17) Treatment of (−)-Nicotine Toxicity by Potencies of a (+)-Nicotine Salt: Experimental Studies on Vibrio fischeri by Testing Bioluminescence Inhibition [20]**

*Vibrio fischeri* was used as test organism to measure the growth inhibitory effect of (−)-nicotine in the presence of (+)-nicotine potencies and placebo. The nicotine experiments in this paper were unique in that a salt of the enantiomer was used in order to prepare potency rather than the enantiomer itself. Specifically the salt used for potency preparation was (+)-nicotine, (+)-di-*p*-toluoyltartrate salt. This salt was used to make individual 4th, 12th, and 30th potencies and also a potency chord consisting of a mixture of these potencies. The toxic effect on bioluminescence of *Vibrio fischeri* was measured. The 4th, 12th, 30th and potency chord potency preparations represent nicotine concentrations of 1.17 × 10^−9^ M, 7.65 × 10^−29^ M, 5.25 × 10^−72^ M and 1.56 × 10^−12^ M, respectively.

#### 5.2.1. *Vibrio fischeri* Bioassay

The test is an acute toxicity test with the marine luminescent bacterium *Vibrio fischeri* (formerly known as *Photobacterium phosphoreum*). *Vibrio fischeri* is a facultative anaerobic, rod-shaped bacterium with a polar whip-like filament or flagellum. The test system measures the light emission of the luminescent bacteria after they have been challenged by a substance or environmental sample and compares it to the light output of a blank control. The difference in intensity of luminescent light emission (between the sample and the control) is due to the effect of the substance/environmental sample on the organisms.

The *Vibrio fischeri* bioassay uses a suspension of *V. fischeri* bacteria in saline water and measures the reduction in light intensity of its natural luminescence on exposure to the toxicant/toxic sample of interest. The bacterial inoculum for the test suspension is prepared under standardised conditions. EC_20_ and EC_50_ values were determined from concentration-response curve after sigmoidal fitting by ORIGIN 6.0 software. EC_20_ = 622 ppm, EC_50_ = 1284 ppm. *Vibrio fischeri* is much more sensitive to nicotine than aspergillus in experiment 24.

**Figure 19:**
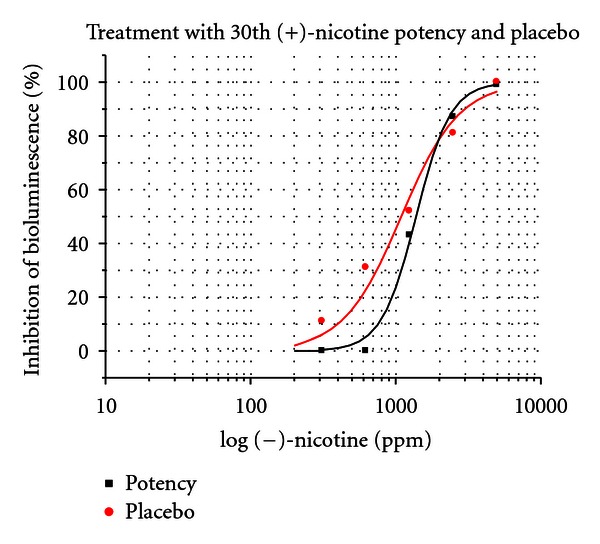
The effect of treatment with 30th potency and placebo on the toxicity of (−)-nicotine. EC_20_ and EC_50_ were significant at the 5% level.

**Figure 20:**
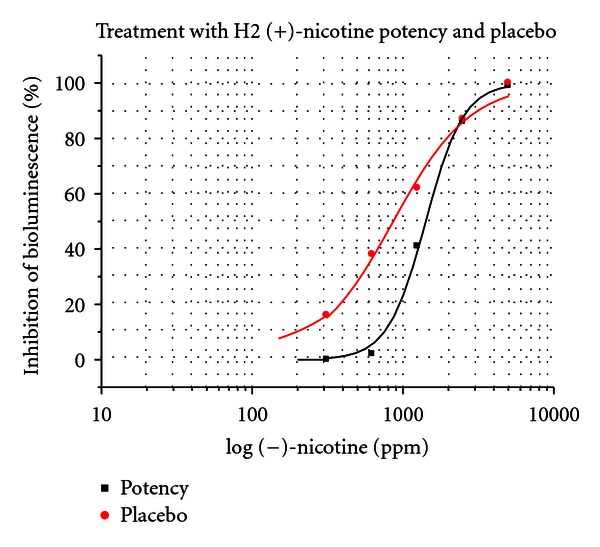
The effect of a potency chord of 4th, 12th, and 30th potencies (H2) of (+)-nicotine salt on toxicity of (−)-nicotine toxicity. EC_20_ and EC_50_ were significant at the 5% level.

The effects of 4th, 12th, and 30th potencies of (+)-nicotine, (+)-di-*p*-toluoyltartrate salt on toxicity of (−)-nicotine at concentrations of 0, 312.5, 625, 1250, 2500, 5000, and 10,000 ppm were tested in 4 separate controlled experiments. In addition the toxicity inhibiting effect of a potency chord consisting of equal parts of 4th, 12th, and 30th potencies was tested. The results were reported by the assayists as significant or insignificant at the 5% level. 4th and 12th potencies were not significantly different from placebo in terms of their effect on (−)-nicotine toxicity. The ultradilute 30th potency and also the potency chord were significantly different from placebo at EC_20_ and EC_50_ at the 5% level. See Figures 19 and 20.

EC_20_ and EC_50_ values were determined from concentration-response curves (Figure 19) after sigmoidal fitting by ORIGIN 6.0 software. Changes in EC_20_ and EC_50_ for Figure 19 are shown in Table 1.

Changes in EC_20_ and EC_50_ for Figure 20 are shown in Table 2.

**(18) Treatment of (−)-Nicotine Toxicity by Potencies of (+)-Nicotine Salt: Experimental Studies on Daphnia magna (the Water flea) [19]**

The *Daphnia magna* immobilization test was considered for testing the toxicity inhibitory effect of (+)-nicotine potency chord on (−)-nicotine toxicity. This is a well-known bioassay [35]. While determining the dose-response curve, it transpired that the sensitivity of *Daphnia magna* to nicotine varied with time after commencement of the test. Although nicotine is very toxic to humans, it was difficult to find organisms for use in biosensors which were sensitive to nicotine. Finally it was decided to use *Vibrio fischeri* and *Daphnia magna*. Other organisms were tested but were rejected due to lack of sensitivity to nicotine. These were *E coli*, *Pseudomonas fluorescens, Tetrahymena pyriformis,* and *Folsomia candida*. *Aspergillus awamori* in experiment 24 needed a high concentration of nicotine to achieve cell death, and this suggests that *Aspergillus* was not an optimal organism for testing toxicity inhibitory effect of potentized (+)-nicotine on toxic doses of (−)-nicotine.

*Daphnia* had maximum sensitivity to nicotine at 72 hours after the test. Subsequently when the hypothesis was tested at the 72-hour mark, the assayist reported that there was a significant decrease in toxicity in the treatment arm at the 5% level. The nicotine concentration of H2 potency chord used in this experiment was 1.56 × 10^−12^ M which is the same as in experiment 17.

### 5.3. Experiments with *Aspergillus awamori*

**Figure 21:**
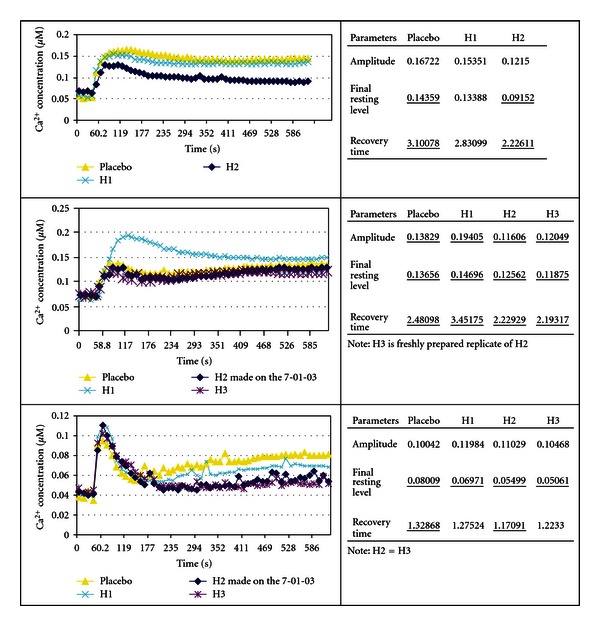
The 3 graphs above, going from top to bottom, show experiments 19, 20, and 21. They show the effect of homeopathic preparations of (R)-(+)-*α*-methylbenzyl isocyanate on [Ca^2+^]_c_ response to 50 mg/L (S)-(−)-*α*-methylbenzyl isocyanate. Underlined data represent statistically significant data at the 5% level. Primary endpoint is final resting level (FRL). Note H1, H2, and H3 homeopathic preparations. H2 was made for experiment 19 and was then kept and used in experiments 20 and 21. H3 represents a fresh potency replicate of H2 made before 9 am on each day of the experiment. Other than the H2 potency made in the isocyanate experiments, all other potencies were made fresh on each day.

The following screening tests were done in Edinburgh, Scotland, in collaboration with Lutess Ltd using *Aspergillus awamori* transformed with the aequorin gene, in another attempt to prove the null hypothesis. This biosensor uses a calcium-sensitive photoprotein, aequorin, which emits light in a dose-dependent manner when bound to free ionized calcium. Higher luminescence means higher concentration of intracellular-free calcium ions. This in turn indicates that the cell is perturbed. Higher luminescence means higher toxicity of the compound. For instance, the ability of treated arms to maintain more normal intracellular calcium compared with the placebo arms reflected in the final resting level (FRL) as shown in the 3 graphs in Figure 21 indicates that the cellular energy consuming transport mechanisms that pump calcium out of cells against extremely high electrochemical gradients are more intact compared with placebo-treated cells [36]. The recovery pathways must be extremely active because they have to work against a 10000 : 1 electrochemical gradient of the Ca^2+^ across membranes [37].

Parameters measured were amplitude (*A*), which is the maximum increase in [Ca^2+^]_c_, achieved after toxic challenge; final resting level (FRL), which is the FRL of [Ca^2+^]_c_ at the end of the experiment, and recovery time (RT), which is luminescence integrated from the point when amplitude is maximal, to the point when [Ca^2+^]_c_ reaches its final resting level at the end of the experiment [32, 36]. FRL was the prospective primary endpoint, and A and RT were secondary endpoints for these experiments.

**Figure 22:**
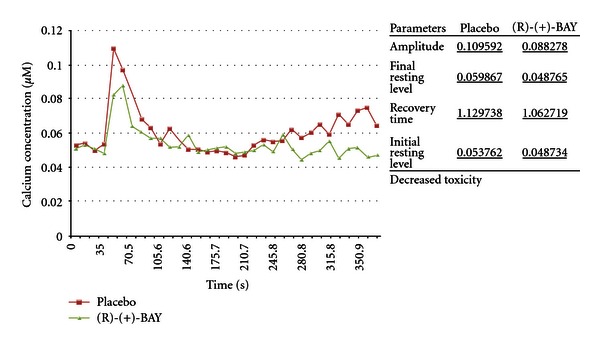
Effect of preincubation of *Aspergillus* with 10 *μ*M (R)-(+)-BAY K8644 on Ca^2+^ response to 10 *μ*M (S)-(−)-BAY K8644. Note that underlined data represent statistically significant data according to the 5% LSD (least significant difference) where *P* = 0.05. Primary endpoint is final resting level (FRL), which in this experiment was measured at 5 minutes.

**Figure 23:**
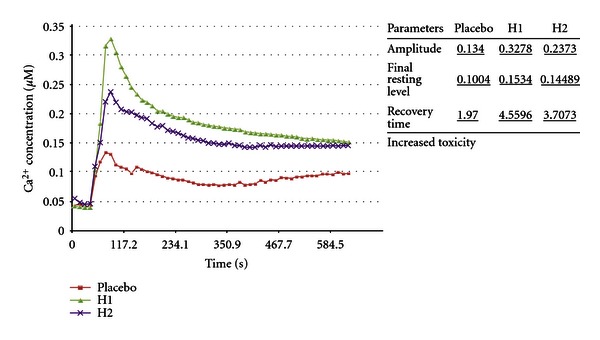
Effect of homeopathic preparations of mixtures of (R)-(+)-*α*-methylbenzyl isocyanate and (S)-(−)-*α*-methylbenzyl isocyanate potencies on [Ca^2+^]_c_ response to 50 mg/L racemic *α*-methylbenzyl isocyanate. Note that H1 and H2 = homeopathic preparations. Underlined data represent statistically significant data at the 5% level. Primary endpoint is final resting level (FRL).

**Figure 24:**
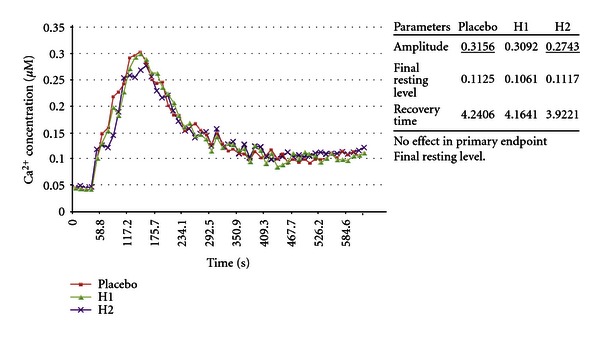
(R)-(+)-nicotine (+)-di-*p*-toluoyltartrate salt and (S)-(−)-nicotine Effect of homeopathic preparations of (R)-(+)-nicotine on [Ca^2+^]_c_ response to 0.5% (S)-(−)-nicotine. Note: H1 and H2 homeopathic preparations. Underlined data represent statistically significant data according to the 5% LSD (least significant difference) where *P* = 0.05. This was only noted in the secondary endpoint Amplitude. Primary endpoint is final resting level (FRL).

**Figure 25:**
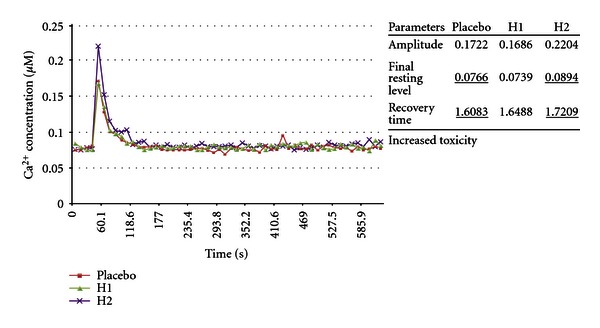
Effect of homeopathic preparations of (R)-(+)-BAY K8644 on [Ca^2+^]_c_ response to 30 *μ*M (S)-(−)-BAY K8644. Note that H1 and H2 homeopathic preparations. Underlined data represent statistically significant data according to the 5% LSD (least significant difference) where *P* = 0.05.

**Figure 26:**
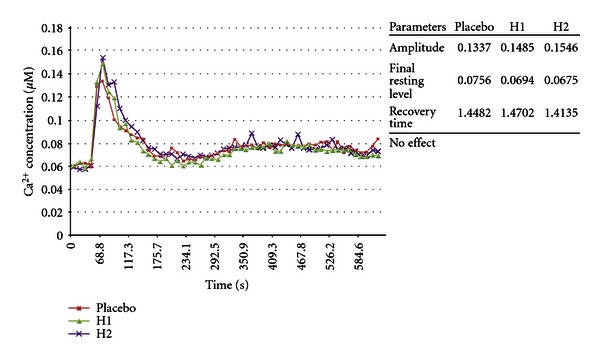
(R)-(+)-verapamil and (S)-(−)-verapamil. Effect of homeopathic preparations of (R)-(+)-verapamil on [Ca^2+^]_c_ response to 0.33 mM (S)-(−)-verapamil. Note that H1 and H2 homeopathic preparations. No effect was found.

Experiments 19–24 inclusive relate to fungal tests, and the first 3 of these, experiments 19–21, test the method using the optical isomer *α*-methylbenzyl isocyanate. The biosensor is described in previous literature [32, 36, 38]. The underlined results in Figures 21–26 show statistically significant differences in toxicity response compared with placebo as reported by Lutess Ltd, Edinburgh. Fungi were cultured in microtitre plates under standardized conditions. After 3 pretreatment doses of homeopathic potency or indistinguishable succussed placebo, the plates were placed in a luminometer. In the *Aspergillus awamori* biosensor tests, the toxin administration and subsequent measurements are fully computerized, and the tests occurred over 10 minutes. During that time each well containing fungal culture on the microtitre plate had over 40 individual measurements.

Each concentration of a toxicant produces a unique [Ca^2+^]_c_ transient [32, 36]. Examples of these are shown in graphs accompanying experiments 19–26. Additionally each concentration of a toxicant produces a unique [Ca^2+^]_c_ transient. Signalling information can be encoded in different characteristics of the “Ca^2+^ signature” such as amplitude (*A*), recovery time (RT), and final resting level (FRL). By mimicking parts of the [Ca^2+^] transient it has been shown that different components of the transient are responsible *in vivo* for protein translocation, protein phosphorylation and gene expression [32, 36, 38, 39]. It is not to be expected that all the endpoints, final resting level (FRL), amplitude (*A*), and recovery time (RT), should respond uniformly for all compounds. Different compounds will affect the toxicity endpoints, Amplitude, final resting level, and Recovery Times in specific ways. For instance, *α*-methylbenzyl isocyanate decreases toxicity as measured by final resting level in 3 experiments in the H2 preparation at the 5% level. This was the prospective endpoint. Another compound may not have significant influence on the final resting level but may have an effect or a consistent effect on another of the toxicity endpoints measured by the methodology. There is no doubt that this method measures toxic effects in a reproducible way [32].

In the case of these experiments, although 30th potency is commonly used in homeopathic research, it is common knowledge that results of experiments may fluctuate and successive experiments may have difficulties with reproducibility. Negative or contradictory results could therefore cast aspersions on the experimental method such that one may suspect the experimental method itself is flawed, and this has occurred in the past [40]. To protect against negative results, we simultaneously tested potency chords consisting of the 4th, 12th, and 30th potencies of the medicinal compound we were testing. The author is not aware of this having been done in the past. The simultaneous or contemporaneous testing of simplexes (single potencies) and mixtures of potencies (potency chords) then continued in the remainder of the experiments.

**(19–21) Modulation of Optical Isomer Toxicity Using Potentized Enantiomers according to an Aspergillus awamori Biosensor: The Example of *α*-methylbenzyl Isocyanate [2, 41]**

Results of experiments 19, 20, and 21 are shown in Figure 21. The ability of potencies of the (+)-isomer of *α*-methylbenzyl isocyanate was tested in terms of their ability to modulate the toxicity of 50 mg/L (S)-(−)-*α*-methylbenzyl isocyanate.

The results under the headings Amplitude, final resting level, and Recovery Time are shown as columns of 3 results each. The first, reading from top to bottom, is placebo, the second is 30th potency, and the 3rd was a potency chord. The consistency noted with the potency chords depicted with H2 and H3 notations was not seen with the single potency preparations depicted by H1. Note in Figure 21 the consistent outcome of the prospective endpoint in 3 consecutive morning experiments with *α*-methylbenzyl isocyanate. Enantiomeric treatment produced consistent decrease in toxicity.

In Figure 21, going from top to bottom, of interest is that the first graph shows that the 30th potency (H1) had no effect. For H1 in the next graph there was an increase in toxicity, and in the third graph in Figure 21 there was a decrease in toxicity. In the two lower graphs in Figure 21, H3 was a freshly made replicate of H2 made on successive days in the morning. H2 and H3 are potency chords of 4th, 12th, and 30th potencies. In other words, they are mixtures of potencies. It has been previously claimed that potency chords produce more consistent responses than single potencies [42, 43].

The observed variability of 30th potency effect is an example of the variable effects observed in homeopathic research and discussed by Walach et al. [44]*;* however, the potency chord responses in the replications showed a consistent decrease in toxicity compared with placebo. This suggests that the fluctuation in potency effect is a property of potency itself and not the biosensor assay method. Factors which may affect potency activity are mentioned in the discussion section.

The data show results, which indicate that stereoisomer potencies may beneficially be used to treat toxicity of *α*-methylbenzyl isocyanate as reported by Lutess Ltd. According to the assayist, this shows consistency of potency chord effect compared with fluctuating 30th potency effect. In the experiments with isocyanate, *α*-methylbenzyl isocyanate with molecular weight 147.17 amu and density 1.045 g/mL at 20°C was used to produce a 30th potency with notional concentration of 4.3 × 10^−67^ M. Potency chord had a concentration of 1.02 × 10^−10^ M.

**(22) Modulation of (S)-(−)-BAY K8644 by (R)-(+)-BAY K8644 [2, 41]**

Results presented in Figure 22 show that preincubation with 10 *μ*M (R)-(+)-BAY K8644 caused a significant inhibition of the [Ca^2+^]_c_ response. This experiment used a concentration of (+)-BAY K8644 in the hormetic range below the “no observed adverse effect level” (NOAEL). The suitable concentration was determined by range finder. Although the concentrations of the enantiomers used were identical, the enantiomers are not equipotent. This is well known. For instance, the (−)-isomer has previously been found to be 10–50 times more potent than the (+)-isomer in terms of effects on calcium channels as shown by effects on vascular smooth muscle and inotropic effects [45]. This experiment tests the principle of hormesis which is elaborated in the discussion.

In addition to the experiments in *Aspergillus awamori* using *α*-methylbenzyl isocyanate referred to above, further experiments were done as shown below screening for the effect of potentized preparations. The original figures show some errors in the tabulated data which appear on the right side of each graph, and these have been corrected in what follows and graphs and tables correlate. The corrected data relate to secondary endpoints and do not affect the primary endpoint “final resting level”. Mainly affected were figures for Amplitude such that some of the numerical data did not correspond to the graphed data. The actual graphs were not affected.

**(23) Modulation of Racemic Optical Isomer Toxicity Using Potentized Enantiomers in an Aspergillus awamori Biosensor: The Example of *α*-methylbenzyl Isocyanate [2, 41]**

This experiment produced a marked increase in toxicity using a unique method of preparation. A mixture of potencies of (R)-(+)-*α*-methylbenzyl isocyanate and (S)-(−)-*α*-methylbenzyl isocyanate caused significant increase in [Ca^2+^]_c_ response compared with placebo. Both H1 and H2 showed increased toxicity in the primary endpoint FRL. Toxicity was also increased in secondary endpoints (Figure 23).

This was an experiment where *α*-methylbenzyl isocyanate was treated by potentized preparations consisting of 50 : 50 mixtures of potencies of both the (+)- and (−)-isomers of the isocyanate, which were prepared independently and then mixed. This experiment was unique, and it was associated with the largest difference between placebo and treatment calcium concentrations of any of the *Aspergillus* biosensor experiments. Further, it was associated with a large increase in toxicity in both the 30th potency and the potency chord, which was against the trend of the other experiments. Mixing potencies of enantiomers produced this unexpected change. In this experiment, *α*-methylbenzyl isocyanate with mw 147.17 and density 1.045 g/mL at 20°C was used to produce a 30th potency of each enantiomer notional concentration of 4.3 × 10^−67^ M. Potency chords of each enantiomer had a concentration of 1.02 × 10^−10^ M in the final solutions.

**(24) Treatment of Toxicity of 0.5% (S)-(−)-nicotine with Potencies of (R)-(+)-nicotine (+)-di-p-Toluoyltartrate Salt [2, 41]**

Experiments 17, 18, and 24 with nicotine are the only experiments using a potency of a salt of the enantiomer of the toxic optical isomer rather than the enantiomer itself. In this case the potency was made using the toluoyltartrate salt of (+)-nicotine. Experiment 24 did not demonstrate a significant effect in the prospective endpoint FRL; secondary endpoints suggested possible effect. Note that 0.5% nicotine is a high concentration of nicotine. This is a 1 : 200 concentration. In comparison, the EC_50_ of (−)-nicotine in the *Vibrio fischeri* test in experiment 24 is only about 1250 ppm, and *Daphnia magna* in experiment 18 was tested in the range 0–2500 ppb. About 3 drops of nicotine rubbed onto the skin of an adult human are enough to cause death.

Results for experiment 24 are shown in Figure 24. If one were to repeat this experiment, then according to the present data, either Amplitude would perhaps be the appropriate prospective endpoint rather than FRL.

Given the high concentration of nicotine which was required in this test, it may be well that *Aspergillus* is not an optimal organism in which to test the effects of nicotine toxicity. Since the investigators were investigating a number of new compounds under specific experimental trophic conditions, they could not be certain which endpoints would be part of the signature for any given compound. In this experiment, the starting solution of 0.5% (R)-(+)-nicotine (+)-di-*p*-toluoyltartrate salt was diluted by a factor of 6.07 × 10^68^. In the case of the potency chord, the dilution was by a factor of 1.44 × 10^11^.

**(25) Aggravation of 30 *μ*M (S)-(−)-BAY K8644 Toxicity by 30th Potency and Potency Chord of (R)-(+)-BAY K8644 [2, 41]**

This experiment reported aggravation of (S)-BAY K8644 toxicity by a potency chord consisting of a mixture of 4th, 12th, and 30th potencies of (R)-(+)-BAY K8644. Potencies were made starting with a 2.8 mM solution of (R)-(+)-BAY K8644. The 30th potency showed no effect on toxicity of the (−)-enantiomer of BAY K8644; however, the potency chord manufactured from (+)-enantiomer was associated with an increase in toxicity of the (−)-enantiomer in the primary end point, FRL (Figure 25). This was the only instance where potency chord was associated with increase in toxicity with this type of manufacture. Note that experiment 23 also produced increased toxicity, but this was using a different and unique method of manufacture. The 30th potency represents a notional concentration of 1.7 × 10^−70^ M. The potency chord represents a BAY-K8644 concentration of 4.04 × 10^−14^ M.

**(26) Modulation of 0.33 mM (S)-(−)-Verapamil HCl Toxicity by Potencies of (R)-(+)-Verapamil HCl [2, 41]**

Verapamil HCl showed no effect in this screening test of either the 30th potency or the potency chord of (R)-(+)-verapamil HCl in the prospective final resting level endpoint. Results are shown in Figure 26. Note that this experiment and the experiment involving nicotine in Figure 24 were accidentally performed with up to 5-hour-old potency in water, whereas protocol dictated use of fresh potencies in water prepared immediately prior to testing, which was the case with the earlier experiments. It is not known if homeopathic potencies in water are stable and, if so, for how long. The experiment needs replication to answer this question. The (R)-(+)-verapamil HCl potencies were made from 10 mM (R)-(+)-verapamil HCl. The 30th potency represented a notional verapamil concentration of 6.05 × 10^−70^ M; the potency chord had a verapamil concentration of 1.44 × 10^−13^ M.

## 6. Discussion

The problem of reproducibility of experiments in homeopathy is described by Walach et al. [44]. Obstacles to reproducibility are discussed by Baumgartner [46]. Walach et al. described the situation: “…What we see in these [*homeopathic*] studies has been a recurrent pattern in homeopathy research, with equally intensive attempts at finding stable *in vitro* models. This pattern is that there are dramatic results, often in the first experimental tests, which are not compatible with random fluctuations only. These results are often also replicable to a certain degree. But rarely are they stable to the degree demanded by mainstream research to accept such phenomena beyond doubt and without a comprehensive theoretical framework…” [44].

Incorrect prescribing in homeopathic practice is discussed by Wiegant et al. [47]. Wiegant and colleagues described features that can be incorporated into study designs, that increase the likelihood that patients randomized to treatment arms are actually receiving correct prescriptions, and the authors provide examples of where this has been done. Otherwise, it is not clear if a negative study has provided evidence of the nonefficacy of homeopathic potencies or is simply underpowered to detect an effect due to incorrect homeopathic prescriptions. Such studies may then find their way into reviews or meta-analyses.

Inherent to the story related in the introduction is that the method of case analysis has a big effect on clinical outcomes in homeopathy. It is optimistic to believe that just because homeopaths have had 5, 10, or 20 years experience, and are professional members of a homeopathic society or group, their prescriptions are necessarily correct. This is certainly contrary to the author's clinical experience, yet it is a basis for selection of treatments in clinical studies of homeopathy [48, 49]. Although these authors did make some attempt at individualization of prescription, one wonders if these attempts were sufficient. It is not difficult to find further examples of this in the literature with even less evidence of individualization of prescription and therefore less reason to believe prescriptions were homeopathic; however, the author does not want to labour the point. Accordingly, power calculations could take incorrect prescribing into account. Yet further, power calculations could consider a suitable noncompliance rate by patient participants.

Another point relevant to correct prescribing in homeopathy is individualization of prescription. One wonders if clinical studies using 10 or fewer remedies in a sample population of 60 patients have been adequately individualized [48, 49]. One study used 29 different remedies for 36 patients [14]. One could counter that the latter study included patients with multiple diagnoses, whereas the other studies focussed on migraines [48] and warts [49]. However, this stance fails to understand that homeopathic diagnosis is not determined by conventional medical diagnoses, rather, by specific symptomatology quite alien to conventional medical diagnosis. One need only to look at a classic homeopathic repertory like Kent in order to observe this [50]. It is quite consistent with the process of homeopathic diagnosis that 100 patients with the same medical diagnosis could conceivably need 100 different homeopathic remedies, whereas 100 patients with different medical diagnoses could, in theory at least, need only several remedies, or even just one remedy. This would be an extreme scenario; however, it serves to illustrate a point.

Regarding selection of suitable experimental models for testing enantiomer potencies, it is suboptimal to test for the ability of a homeopathic optical isomer to inhibit toxicity of its enantiomer, if the enantiomer in question is either not toxic or minimally toxic to the organism one is using in a test system. If too high a concentration of toxin is needed, then this can have confounding physicochemical or biological effects, for example, denaturation of proteins or changes in pH. Similarly, there is no point testing the null hypothesis if the test method is not functioning within normal parameters or margins of error. As stated by one collaborator, no single toxicity test can be used to test the effects of toxicants in all biota [32].

All but 5 experimental series reported here provided evidence of decrease in toxicity when using pairs of enantiomers according to prospective criteria. One of these, experiment 23, reported increased toxicity, and 4 experiments reported no evidence of effect in the primary endpoint but pointed to possible effect in secondary endpoints: experiments 7, 8, 24 and 26. The relevance of these findings would require dedicated prospective experiments to confirm. Experiments 24, and 26 used 4-5-hour-old potencies in water which was contrary to the preceding experiments' protocol, and it is not known for how long potencies are stable in water.

Experiment 15 provided an interesting result in the maximal treatment arm depicted by the line at the top of the legend of Figure 15; however, the experiment suffered from no stratification and too many treatment arms. It was noted that in no case in the *Tetrahymena* experiments did a placebo arm poisoned with glutamic acid have increased survival compared with the corresponding treatment arm(s).

Current evidence is that use of homeopathic simplex potencies in enantiomeric treatment produces fluctuating effects, as exemplified in Figure 21. In other words, if replications of experiments, say, with *Vibrio fischeri *are performed using single potencies (also called simplexes), then as has been the case in previous research in homeopathy, we would expect to see evidence of the variability of outcome described by Walach et al. [44] and also recently by Endler et al. [51].

In Figure 21 we see the example of (−)-*α*-methylbenzyl isocyanate (see experiments 19–21); even though the effect of treatment with 30th potency (+)-*α*-methylbenzyl isocyanate fluctuated in sequential experiments conducted in the morning on different days, the outcome of simultaneously conducted potency chord experiments using the (+)-isomer showed consistent decrease in toxicity of the *α*-methylbenzyl isocyanate. The consistency of effect of the experiments using potency chords provided reassurance that the cause of the fluctuation in effect of 30th potency was due to a confounding factor influencing the efficacy of the 30th potency, and not due to inconsistency of the experimental method. This is a type of “positive control” which can be considered as a possibility together with systematic negative controls in homeopathic research that are mentioned by Baumgartner [46].

A consistent inhibitory effect of simultaneously tested potency chords and individual potencies (simplexes), such as the commonly used 30th potency, in an experimental model can provide another layer of reassurance that fluctuating effects of simplexes such as the 30th potency which is commonly used in homeopathy research are not due to systematic or stochastic errors of the test system referred to by Baumgartner [46] but rather are due to the inherent properties of the single potencies themselves. This was most obviously demonstrated in the case of the isocyanate experiments in this paper shown in Figure 21.

Are homeopathic potencies simple dilutions? Is there any difference between a 4th centesimal potency representing an attenuation of by a factor of 10^8^ or a dilution prepared as in hormesis, by simply dissolving a small amount of substance in sufficient diluents such that an attenuation of the same magnitude results? Hormesis describes dose-response relationships characterized by a reversal of response between low and high doses of chemicals, biological molecules, physical stressors, or other initiators of a response [52].

For dilutions used in hormesis, where succussion is not the norm, the concentration range at which effects are observed is only between about 1/5th–1/100th the dose of the “no observed adverse effect level” (NOAEL) in toxicology and more commonly 1/5th–1/10th the dose of the NOAEL [53, 54]. In homeopathy, effects are reported well below this right up to the ultramolecular range that is, dilutions beyond Avogadro's number. Such results are also reported in the present paper. This suggests that solutions prepared by serial dilution and agitation as in homeopathy are indeed different from simple dilutions, that is, solutions which have not undergone serial dilution and succussion or other form of agitation.

Another point of note is to consider the actual manufacture of potency, say, the 30th centesimal potency produced in ethanol by serial dilution and succussion. This represents an attenuation of 10^−60^. This is well beyond Avogadro's number. Some of the attenuations used in optical isomers have been in this range. Such a solution can be comfortably made within 45 minutes. To make a 30th centesimal potency would require dilution of 0.1 gm medicinal substance into 10^56^ litres of diluent. If this were contained in a single test tube with 1 mL graduations/cm, the test tube would be 10^54^ km in length. To evenly disperse 0.1 gm of soluble medicinal substance in such a test tube within 45 minutes (the time in which one could manufacture a notional 10^60^ attenuation in homeopathy) would in theory need particles of the medicinal substance to travel at greater than the approximately 300,000 km/s speed of light. Also from this perspective the homeopathic attenuation does not seem to a simple dilution.

The 30th potency is widely used in homeopathic research, and the variability of effect has been discussed [44, 46]. Nonreproducible effects may occur when the natural laws governing a field are inadequately known, and hence it is possible that a range of factors such as electrostatic effects, humidity, optimal production, and storage conditions may be interfering with reproducibility of experiments [46]. Scant attention has been given to a possible role for gravitation and geodesics in modulating action of homeopathic potencies during manufacture. Given the potential for such variability of effect of potencies in homeopathy, it is not surprising that homeopathic research has had difficulty obtaining consistent effects when using homeopathic remedies made from single potencies or dilutions. The experiments using isocyanate reviewed in Figure 21 were done on different days. The 30th potencies varied markedly in effect compared with the potency chords. There are numerous factors which may impact on potency activity which affect the individual potency, but which the mixture of potencies (potency chord) may at least partially protect against.

In experiment 23 an unexpected outcome was observed when toxicity of the isocyanate was treated with a potency made by mixing enantiomers. In this case a marked increase in toxicity was observed. Testing mixtures of potencies in rapid succussion were actually attempted in 2006. Multiple microtitre plates were tested in quick succussion in an attempt to collect data rapidly involving various different compounds and potency preparations and mixtures. The data proved to be uninterpretable due to a large and atypical increase in variance of luminometer measurements by a factor of several times resulting in chaotic measurements. One wonders if mixing of potencies of enantiomers of the same or different substances may cause a situation of deterministic chaos as described in chaos theory. Alternatively, the wide variances may simply have been related to a faulty batch of aspergillus used in the assay or other deviation in protocol. Mixing may have occurred between experiments by condensation in the measuring chamber of the luminometer used in the experiments.

The use of biosensors or other rapid methods for testing potency activity may have a role in pretesting homeopathic potencies used in clinical research. Prior to performing large expensive and time-consuming studies on patients, it would be reassuring to confirm bioactivity of the test solutions prior to commencing the clinical studies.

A reproducible model for testing potencies will allow the investigation of other basic questions in potency research. These include, but are not limited to, the investigation of the efficacy of succussed versus nonsuccussed solutions, the effects of gravitation or geodesics, and the stability of potencies in water; that is, if potencies in water are bioactive, then how long are they bioactive for? This has obvious practical consequences in the design of experiments. If a test system uses water potencies, then it begs the question that a negative result is necessarily due to nonefficacy of the treatment, since it may also be due to loss of bioactivity of the water potencies.

The level of evidence provided by each experiment contained herein varied; however, diverse methods, molecules, and species provide a web of mutually confirmatory data. Each of the experimental series tested the same hypothesis related to optical isomer modulation by potencies of their enantiomers using prospective designs, and results were achieved with minimal or no pretesting for optimal potencies.

### 6.1. Hypothesis-Driven Research Using Enantiomers in Homeopath: The Example of Glutamic Acid

Use of homeopathy in mood-related disorders has recently been reviewed [55]. A possible clinical application to consider in use of enantiomeric potencies is the effect of D-glutamic acid potencies on inhibition of L-glutamic acid neurotoxicity with a view to improving symptoms of anxiety, low mood and other L-glutamic acid-related conditions.

Glutamate is the major excitatory synaptic neurotransmitter in the brain and is found in 80% of neurons, and there is increasing evidence that antagonists of glutamate action at NMDA receptors have antidepressant-like action and anxiolytic action [56–59]. Glutamate is reported to be largely responsible for the ability of the nervous system to rapidly transmit information from one part of the body to another, and to be important in thought formation and memories [60]. Although most CNS glutamate is synthesized in the CNS, it crosses the blood-brain barrier at a rate of 0.67 nmol min^−1^ g^−1^ [61].

The term excitotoxicity refers to the excessive exposure to the neurotransmitter glutamate or to stimulation of its membrane receptors and is considered a main contributor to neuronal injury and death in numerous conditions [60]. In the case of the NMDA receptors these include Alzheimer's disease, Parkinson's disease, Huntington's disease, HIV-associated dementia, multiple sclerosis, amyotrophic lateral sclerosis, and glaucoma, obsessive-compulsive disorder, stroke, dementia and neuropathic pain [60], and also anxiety and depression [57, 59, 62, 63].

Another group of glutamate receptors is the metabotropic glutamate receptors. Evidence suggests that inhibition of some receptors belonging to this category may play a part in counteracting nicotine addiction; other receptors in the same category may assist with depression occurring in early nicotine withdrawal and may be useful in treatment of depression generally [64]. Metabotropic glutamate receptors may also be promising targets in the treatment for neurologic disorders derived from abused drugs such as cocaine, morphine, and amphetamines and may also play a part in the regulation of several neurodegenerative disorders, epilepsy, and ischemia [65]. Some metabotropic receptor agonists may be useful in the treatment of psychotic disorders including schizophrenia [66–70]. Another approach may be to modulate action of glutamate coagonists glycine and D-serine [70]. As shown in figure 23, use of potentized enantiomers has the potential to increase as well as decrease toxic or physiological action of optical isomers for the purpose of modulating activity.

Other possible uses of potentized enantiomers may be the inhibition of (+)-nicotine toxicity, including addiction, in tobacco smokers, and the inhibition of action of narcotic drugs of addiction such as opiates and as already exemplified using U-50488 in mice; also modulation of activity of optically active coordination compounds involving transition metals [71].

## Figures and Tables

**Table 1:** The effect of (+)-nicotine salt 30th potency on toxicity of (−)-nicotine toxicity—EC_20_ and EC_50_ values.

	EC_20_ (ppm)	EC_50_ (ppm)
30th potency	958	1399
30th placebo	580	1088

**Table 2:** The effect of a potency chord of 4th, 12th, and 30th potencies of (+)-nicotine salt on toxicity of (−)-nicotine toxicity—EC_20_ and EC_50_ values.

	EC_20_ (ppm)	EC_50_ (ppm)
H2 potency	958	1423
H2 placebo	382	864
